# Editorial: Nanobody-based cancer immunotherapy and immunoimaging

**DOI:** 10.3389/fimmu.2023.1213386

**Published:** 2023-05-16

**Authors:** Mahdie Jafari, Amirhosein Maali, Mohammad Ali Shokrgozar, Zahra Sharifzadeh

**Affiliations:** ^1^ Department of Immunology, Pasteur Institute of Iran, Tehran, Iran; ^2^ National Cell Bank of Iran, Pasteur Institute of Iran, Tehran, Iran

**Keywords:** nanobodies, single domain antibodies, cancer, immunotherapy, immunoimaging

Nanobody, the single domain antibody fragment derived from camelid antibodies, is known as the smallest naturally occurring antibody domain capable to attach to antigens with higher affinity and specificity compared to conventional antibodies (1). Considering their excellent properties, nanobodies have been extensively used in the field of immunotherapy and immunoimaging, especially for cancer diseases (2). The smaller size of nanobodies, compared to other antibody formats, enables them to efficiently penetrate into the tumor site which in turn provides more accessibility to tumor cells for therapeutics and imaging agents. Also, their remarkable stability and solubility together with reduced immunogenicity makes them ideal candidates for tumor targeting (3).

Nanobodies have been used to target cancer cells trough binding to specific cancer antigens. However, they are unable to trigger nanobody-mediated antibody-dependent cellular cytotoxicity (ADCC) and complement-dependent cytotoxicity (CDC) due the the lack of Fc domain. Zhong et al. addressed this problem through fusing a nanobody targeting Claudin18.2 antigen with human IgG1 Fc. They showed the promising therapeutic potential of this humanized nanobody/IgG1-Fc fusion protein on Caludin18.2-positive cancer cells through ADCC and CDC. Moreover, the anti-Caludin18.2 fusion nanobody showed better tumor penetration and faster tumor uptake than a chimeric IgG1 monoclonal antibody.

In addition to direct tumor-targeting, nanobodies are exploited in cell-mediated cancer immunotherapy. In this regard, Maali et al. reviewed the application of nanobodies in bridging between tumor and immune cells through bi- and multi-specific T and NK cells’ engagers. Targeting tumor cells with engineered T and NK cells harboring nanobody-based chimeric antigen receptors (CARs) has been discussed as the most successful application of nanobodies in cell-mediated immunotherapy. Also, authors reviewed the different nanobody-based strategies used to enhance anti-tumor functions of macrophages. Finally, the role of nanobodies in reversing the T cell exhaustion and managing the adverse effects of different immune cell therapies is discussed. As a nanobody-based cell therapy, Nasiri et al. successfully developed second generation CAR-T cells to target CD19-positive tumor cells. They showed that the nanobody-redirected CAR-T cells had expansion, cytotoxicity and proinflammatory cytokine secretion rates comparable to those of their scFv-based counterparts.

As mentioned above, nanobodies may act as the blockers of antigens, receptors, or intracellular mediators. In an interesting approach, Demeules et al. studied the *in vivo* purinergic checkpoint inhibition by different specific nanobodies expressed through adeno-associated viral vectors. Nanobody-mediated blocking of P2X7, a ligand-gated cation channel, significantly inhibited tumor growth in P2X7-expressing tumor models. Moreover, a bispecific nanobody-based biologic targeting CD73 (an ecto-enzyme catalyzing extracellular ATP into immunosuppressive adenosine) and PD-L1 successfully inhibited the growth and metastasis of tumor cells. Also, Keller et al. employed the intracellular expression of a nanobody specific to the GTP-bound conformation of RHOA subfamily GTPases to distrupt the RHOA/ROCK signaling pathway. They established that this functional intracellular nanobody resulted in the loss of cellular contraction properties in metastatic melanoma cells which may have implication in cancer therapy. Genetically modifying nanobodies has enabled a new generation of receptor-specific probes that target EGFR. Comez et al. discovered two nanobodies that bind to the same receptor site as EGF and other ligands that bind to EGFR. Using the NanoBRET technology, they could monitor the G protein-coupled receptor ligand binding and conformational changes of EGFR. This study proved the hopeful function of nanobodies for studying the role of the EGFR in health and disease.

Regarding their small size, single domain nature and improved stability, nanobodies represent promising candidates for facilitated delivery to the brain. Zheng et al. reviewed the application of nanobodies as research tools, diagnostic agents and therapies in brain diseases, focusing on brain tumors, Alzheimer’s disease, and Parkinson’s disease. In this study, they provide an overview of the different methods for transportation of nanobodies to the brain. The natural methods of brain-blood-barrier (BBB) penetration include passive diffusion, active efflux, carrier-mediated transport and transcytosis. Other strategies of cerebral delivery of nanobodies employ the structures with the ability to pass the brain as well as some means that temporarily increase the BBB permeability.

CD38 is a tumor antigen which is overexpressed in multiple myeloma, and has emerged as an ideal therapeutic target for cancer therapeutics. Hambach et al. reviewed the application of nanobody-based biologics including heavy chain antibodies, bispecific or trispecific killer cell engagers (BiKEs or TriKEs), CAR-NK cells, and nanobody-displaying adeno-associated viral vectors in efficient targeting of CD38-expressing myeloma cells. Detection of multiple myeloma in patients treated with daratumumab, a CD38-specific monoclonal antibody, is difficult due to the overlapping binding sites of daratumumab and CD38-specific imaging antibodies. Pape et al. developed a nanobody that identifies a unique, non-overlapping epitope on CD38 and labeled it with Alexa Fluor 680. This nanobody could preferentially bind to CD38 on myeloma cells, allowing selective imaging of CD38-expressing xenografts in daratumumab-pretreated mice.

In recent years, oncolytic viruses (Ovs) have emerged as a worthwhile treatment option in cancer therapy. Jafari et al. reviewed the role of combining oncolytic virotherapy and antibody-based therapeutic approaches in cancer. They discussed the benefits of OVs’ combination with antibodies, nanobodies, CAR T cells, and antigen presenting cells to reduce side effects and boost anti-tumor efficacy. The results of ongoing clinical trials can help researchers create innovative combination therapy systems and bring forth ground-breaking treatments for patients. Kadkhodazadeh et al. investigated whether the SpyTag-SpyCatcher system can modulate adenovirus (Ad) tropism and induce covalent virus-adaptor molecule interactions. SpyCatcher was genetically fused with a VEGFR2-specific nanobody to develop a retargeted Ad vector. The recombinant Ad vector, which included a SpyTag peptide in its HI loop, could efficiently target VEGFR2-expressing cells via the primary Ad receptor-independent pathway. The results indicated that this functionalized Ad vector has therapeutic promise for cancer. This viral vector may target additional ligands for theranostic purposes and reduce the hepatotoxicity of systemic Ad delivery.

Considering the advantages of nanobodies and their applications, as well as the research cited regarding the use of nanobodies in the treatment and detection of different diseases, particularly cancer, it can be concluded that nanobodies could definitely be employed as an effective therapeutic and diagnostic agent.

## Author contributions

All authors listed have made a substantial, direct, and intellectual contribution to the work and approved it for publication.
